# Editorial: Lung Ultrasound in the Diagnosis of Infective Lung Diseases

**DOI:** 10.3389/fmed.2022.844590

**Published:** 2022-02-01

**Authors:** Guglielmo M. Trovato, Marco Sperandeo

**Affiliations:** ^1^The European Medical Association, Brussels, Belgium; ^2^University of Catania, Catania, Italy; ^3^Unit of Interventional and Diagnostic Ultrasound, IRCCS Hospital Casa Sollievo della Sofferenza, Foggia, Italy

**Keywords:** pleural effusion, digital health (eHealth), digital medicine, fake news, pneumonitis, COVID-19, guidelines and recommendations, ultrasound

We live in a revolutionary era of medicine focused on digital health. Regretfully, every revolution evaporates and leaves behind only an undesirable new bureaucracy.

Today, too many healthcare professionals, everywhere, are charged full-time with tasks related to accounting, monitoring, vaccination, diagnostics, and therapy of COVID-19 patients. Focus on the Research Topic of Lung Ultrasound in the Diagnosis of Infective Lung Diseases is also a plea for caution, coping with the disruptive impact of misuse of digital imaging medicine amid the COVID-19 pandemic (1).

Lung ultrasound is a niche operator-dependent methodology, which requires accurate and prolonged training, suitable to a role complementary of methods that are more definite.

The clinical presentation of COVID-19 patients, consequent mainly to severe pulmonary complications, is well detected by imaging, even with lung ultrasound. Sadly, sequelae of pulmonary, muscular, neurological, and myocardial involvement are frequent, leading to post-COVID-19 syndrome (2). It is particularly regrettable that in the effort to propose diagnostic and therapeutic approaches, some people have turned to illogical schemes, assertions, and recommendations. In lung ultrasound, the old method of B-lines is resumed. These erratic artifacts are meagerly claimed as pathognomonic changes in pulmonary pathology and even specific for COVID-19 pulmonary disease (3–5). We are witnessing ever more numerous attempts to spread the incongruous use of lung ultrasound, measuring by eye, going at a glance for approximate and erratic counts of the number of B-lines in emergency-urgency subsets and in intensive care.

Indeed, this approach;

1. conceals professional incapacity in carrying out a procedure that has specific areas of implementation and application;
2. produces boastful diagnoses proposed in arbitrary ways;
3. encourages the purchase of ultrasound machines that are entrusted to inexperienced hands for this use, while such equipment would be valuable if used to their full potential by adequately trained health professionals;
4. recommends a cumbersome and useless ritual, crediting it as a diagnostic procedure.

This is precisely the paradigm of a bad use of electronic and digital technology, which ultimately deceives the operator, patients, other doctors, the scientific community, and the committed critical stakeholders, and which has no sustainable basis and which is neither validated nor suitable to be validated.

Elsewhere, experts of all kinds have rapidly occupied the field of big data management in healthcare. Clumsy clinical, experimental, and computational approaches may produce devastating effects, by their mathematical credit, influencing questionable and even contradictory choices of policy makers, decision makers, and opinion leaders. This is one of the reasons for the current mistrust in medicine and science, violent feedback, mass reactions, despair, and unmotivated depression of individuals, even dealing with manageable or solvable problems (6).

Some titles of this special issue itemize the most important achievements of lung ultrasound in pulmonary disease, and their merits and limitations. In Artificial Intelligence (AI) and Lung Ultrasound, Trovato and Russo outline why it is unlikely that the algorithms proposed may directly replace medical doctors, and, namely, the radiologists' judgments as well as personal responsibility during ultrasound diagnosis. In COVID-19 Pneumonia: The Great Ultrasonography Mimicker, Lacedonia et al. thoroughly present the great number of diseases and pathologic conditions that may mimic COVID-19 pneumonia on LUS examination. In not always and not only what is COVID-19 “Glitters”, Quarato et al. demonstrate that LUS is not an adequate tool for screening purposes in the ED, due to the risk of missing some lesions and/or to underestimate the actual extent of the disease. Furthermore, the non-specificity of LUS implies the possibility to erroneously classify pre-existing or overlapping conditions as COVID-19 pneumonia. Transthoracic ultrasound in infectious organizing pneumonia: a useful guide for percutaneous needle biopsy by Lacedonia et al., based on a lasting and large personal experience, outline how, although ultrasound findings did not allow the characterization of chronic subpleural lesions, TUS was confirmed to be a valid diagnostic aid for guiding percutaneous needle biopsy of subpleural consolidations. Lung Ultrasound in Patients With Dyspnea From Infective Lung Disease by Bracciale et al. is a very stimulating article which describes the methodological and standardized use of bedside LUS in the differential diagnosis of patients with acute dyspnea from infective lung diseases. Chest imaging in the diagnosis and management of pulmonary tuberculosis: the complementary role of thoracic ultrasound, a perspective article by Rea et al. focuses on the potential role of TUS in the diagnosis and management of patients with pulmonary tuberculosis.

Surreptitiously “doping” the diagnostic potential of pleural-pulmonary ultrasound in infectious and non-infectious lungs is still attempted, following wishful thinking and strongly believing in the possibility of resolving any diagnostic doubts by naive methods that were claimed as easily repeatable and inexpensive. Therefore, established best and rational practice was not fully pursued. Papers, stacked on top of each other like stackable chairs, were focused on the claimed usefulness of ultrasound artifacts. These are machine or operator errors, and in any case phantasmagorias that do not have an anatomical equivalent. The illogical leap was to try to impose them as potential predictors of disease, despite the fact that for over 30 years it has been well confirmed that these are limits of pleural-pulmonary ultrasound imaging (7). Driven by an exaggerated enthusiasm, it has been hypothesized that the diagnostic value of artifacts is specific to some peculiar diseases, ignoring and never citing all the studies previously carried out. Replicas and imaginative variations in literature were proposed and published, claiming repeatedly unlikely diagnostic novelty. This is a pathway for putting knowledge before wisdom, ad-libbing before skill and intelligence before common sense, treating patients as cases, and making the cure of the disease more painful than enduring it.

Furthermore, algorithms were passed off as innovative and sustainable medicine, optimal for directives, recommendations, or statistics (8, 9). Ethical responsibilities and democratic accountability of researchers in their role as experts and policy advisors are great. We too warn against the potential misuse or misleading interpretation of public data of variable quality and the use of inadequate study designs for the evaluation of effect of non-pharmaceutical interventions (10). We do not need the quest of surrogating clinical medicine by unreliable practices and questionable digital applications; this is mostly relevant if the chosen data source and the methods for achieving them are vague, slippery and inadequate.

## Author Contributions

All authors listed have made a substantial, direct, and intellectual contribution to the work and approved it for publication.

## Conflict of Interest

The authors declare that the research was conducted in the absence of any commercial or financial relationships that could be construed as a potential conflict of interest.

## Publisher's Note

All claims expressed in this article are solely those of the authors and do not necessarily represent those of their affiliated organizations, or those of the publisher, the editors and the reviewers. Any product that may be evaluated in this article, or claim that may be made by its manufacturer, is not guaranteed or endorsed by the publisher.
